# The effect of androgen supplementation on hair loss: A systematic review

**DOI:** 10.1016/j.jdin.2026.03.011

**Published:** 2026-03-27

**Authors:** Sana Gupta, Jia Qi Adam Bai, Ryan S.Q. Geng, Jeffrey Donovan

**Affiliations:** a Faculty of Medicine, University of Ottawa, Ottawa, Ontario, Canada; b Temerty Faculty of Medicine, University of Toronto, Toronto, Ontario, Canada; c Department of Dermatology and Skin Science, University of British Columbia, Vancouver, British Columbia, Canada; d Donovan Hair Clinic, Whistler, British Columbia, Canada

**Keywords:** androgen, androgenetic alopecia, male pattern hair loss, medical dermatology, systematic review

*To the Editor:* Exogenous androgen therapy is increasingly prescribed across diverse patient populations.^1^ Understanding its impact is particularly important for patients with androgenetic alopecia (AGA), an androgen-dependent form of hair loss.^2^^,^^3^ Despite widespread use, effects of androgen supplementation on AGA onset and progression have not been comprehensively reviewed. This systematic review summarizes current evidence on the association between exogenous androgen supplementation and hair loss.

Following Preferred Reporting Items for Systematic Reviews and Meta-Analyses guidelines, MEDLINE, Embase, and CENTRAL were searched in July 2025 (Supplementary Table I, available via Mendeley at https://data.mendeley.com/datasets/z7ncw78mws/1). Fourteen studies with 2344 patients were included (Fig 1). Per Joanna Briggs Institute tool, 10 studies had low risk of bias and 4 moderate risk of bias (Supplementary Table II, available via Mendeley at https://data.mendeley.com/datasets/z7ncw78mws/1).

**Fig 1:**
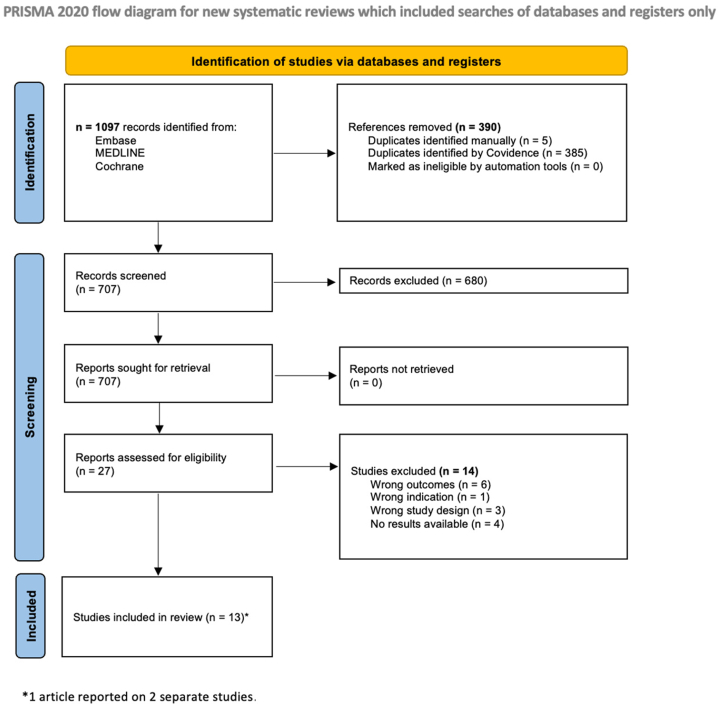
Flow diagram of literature screening using the Preferred Reporting Items for Systematic Reviews and Meta-Analyses (PRISMA) guidelines. Figure adapted from https://www.prisma-statement.org. Eligible studies included English-language primary literature (randomized controlled trials, observational studies, case series ≥5 patients) reporting hair loss outcomes following androgen supplementation. Animal studies, in vitro experiments, case reports, and systematic reviews/meta-analyses were excluded.

Participants were 73.0% female (*n* = 1712/2344; mean age: 33.5 years) and 27.0% male (*n* = 632/2344; mean age: not reported), as assigned at birth (Table I). 25.6% (*n* = 599/2344) reported baseline hair loss, predominantly AGA (86.6%, *n* = 520/599). Indications for androgen supplementation included gender-affirming hormone therapy (59.0%, *n* = 1383/2344), androgen deficiency (12.2%, *n* = 285/2344), and anabolic-androgen steroids (4.3%, *n* = 100/2344). Two experimental trials (*n* = 495) tested androgens as treatment for existing alopecia; 494/495 participants in these trials had AGA. Testosterone and testosterone esters were the most frequently administered androgen (99.8%, *n* = 2340/2344).

**Table I:** Summarized clinical and study characteristics of the included studies

Clinical characteristics (*n* = 2344)	*N* (%)
Age (y), mean (*n* = 299)	33.60
Sex	
Male	632 (27.0)
Female	1712 (73.0)
Baseline hair loss diagnosis (*n* = 1731)	
Androgenetic alopecia	520 (30.0)
Unspecified alopecia	2 (0.1)
Alopecia universalis	1 (0.1)
Supplement indication	
Gender-affirming hormone therapy	1383 (59.0)
Experimental treatment of hair loss	495 (21.1)
Androgen deficiency	285 (12.2)
Anabolic-androgen steroids	100 (4.3)
Supplement duration (wk), mean (*n* = 1776)	49.59
Androgen supplement	
Testosterone	1478 (63.1)
Testosterone propionate	495 (21.1)
Testosterone enanthate/cypionate	166 (7.1)
Testosterone undecanoate	105 (4.5)
Testosterone ester	96 (4.1)
Trenbolone	52 (2.2)
Drostanolone	39 (1.7)
Boldenone	38 (1.6)
Nandrolone	33 (1.4)
Stanozolol	29 (1.2)
Methandienone	25 (1.1)
Oxandrolone	23 (1.0)
Mesterolone	19 (0.8)
Metenolone	17 (0.7)
Oxymetholone	15 (0.6)
Dehydrochlorotestosterone	2 (0.09)
19-Norandrostenedione	1 (0.04)
Testolone	1 (0.04)
Fluoxymesterone	1 (0.04)
Study characteristics (*n* = 14)	
Study design	
Prospective cohort	5 (35.7)
Retrospective cohort	4 (28.6)
Cross-sectional	4 (28.6)
Randomized controlled trial	1 (7.1)
Study population	
Pediatric only	0 (0.0)
Adult only	12 (85.7)
Adult and pediatric	2 (14.3)

Among transgender men receiving androgens for gender-affirming therapy, 19.5% (*n* = 269/1383) developed AGA compared to 1.9% (*n* = 26/1383) at baseline (Supplementary Table III, available via Mendeley at https://data.mendeley.com/datasets/z7ncw78mws/1). Mean treatment duration was 60.7 weeks (range: 12-104) with mean follow-up of 60.9 months (range: 4-119). In studies reporting AGA severity post-therapy, 37.3% (*n* = 50/134) reported moderate to severe hair loss (Hamilton-Norwood score ≥III).

In 285 androgen-deficient females using subcutaneous testosterone, none reported hair loss. Of those reporting hair thinning before testosterone therapy, 63.1% (*n* = 48/76) observed regrowth. Hair loss outcomes in androgen-deficient males were not available. Among 100 male anabolic-androgen steroid users, alopecia prevalence increased from 2.0% (*n* = 2/100) at baseline to 12.0% (*n* = 12/100) after 2 to 52 weeks of use.

Among male participants in experimental trials evaluating use of topical testosterone for pre-existing hair loss (AGA, *n* = 494/495), 16.3% (*n* = 81/495) reported worsening hair loss after 6 weeks. 17.3% (*n* = 86/495) experienced improvement; 66.3% (*n* = 328/495) reported no change.

Adverse events occurred in 23.5% (*n* = 552/2344) of participants using androgens, most commonly acne (5.6%, *n* = 132/2344) and testicular atrophy (9.0%, *n* = 57/632) in males (Supplementary Table IV, available via Mendeley at https://data.mendeley.com/datasets/z7ncw78mws/1). 8 (1.5%) discontinued androgens due to adverse events, including depression (*n* = 1) and distress from AGA (*n* = 1).

Overall, this review highlights the potential risk of hair loss with exogenous androgen therapy across various indications. However, evidence remains heterogenous and hair growth can occur in some settings.^2^ Notably, hair regrowth in androgen-deficient females suggests modified intrinsic follicular responses to restored androgen signaling in this population.^4^ More robust studies are needed to clarify mechanisms in all these settings. Limitations of this review include small sample sizes and self-reported outcomes.

Both medical and nonmedical use of androgens appears to be increasing in the past few decades. Importantly, androgen use continues to extend beyond specialty-care settings, with bioidentical hormone supplementation being marketed for understudied indications including antiaging.^5^ Given limited regulation and potential hair loss risks surrounding these applications, contemporary studies with diverse cohorts and objective measurements can help better assess effects of androgen supplementation.

### Declaration of generative AI and AI-assisted technologies in the writing process

Not applicable.

## Conflicts of interest

Dr Donovan has received honoraria from Pfizer and Vichy, has participated on advisory boards at Pfizer for payment, receives Royalties from UpToDate, participates on the Board of Directors for the Scarring Alopecia Foundation, and is the active Director of the Evidence Based Hair Fellowship Training Program. Sana Gupta, Jia Qi Adam Bai, and Ryan S. Q. Geng have no conflicts of interest to declare.
